# Distributed Sensing Network Enabled by High-Scattering MgO-Doped Optical Fibers for 3D Temperature Monitoring of Thermal Ablation in Liver Phantom

**DOI:** 10.3390/s21030828

**Published:** 2021-01-27

**Authors:** Aidana Beisenova, Aizhan Issatayeva, Zhannat Ashikbayeva, Madina Jelbuldina, Arman Aitkulov, Vassilis Inglezakis, Wilfried Blanc, Paola Saccomandi, Carlo Molardi, Daniele Tosi

**Affiliations:** 1 Department of Computer and Electrical Engineering, Nazarbayev University, Kabanbay batyr, Nur-Sultan 010000, Kazakhstan; aidana.beisenova@nu.edu.kz (A.B.); zhashikbayeva@nu.edu.kz (Z.A.); madina.jelbuldina@nu.edu.kz (M.J.); Arman.Aitkulov@nu.edu.kz (A.A.); carlo.molardi@nu.edu.kz (C.M.); daniele.tosi@nu.edu.kz (D.T.); 2 Laboratory of Biosensors and Bioinstruments, National Laboratory of Astana, Kabanbay batyr, Nur-Sultan 010000, Kazakhstan; 3 Department of Chemical and Process Engineering, University of Strathclyde, 75 Montrose Street, Glasgow G1 1XJ, UK; vasileios.inglezakis@strath.ac.uk; 4 Université Côte d’Azur, INPHYNI, CNRS UMR 7010, Parc Valrose, 06108 Nice, France; wilfried.blanc@inphyni.cnrs.fr; 5 Politechnico di Milano, Department of Mechanical Engineering, Giuseppe La Masa, 20156 Milano, Italy; paola.saccomandi@polimi.it

**Keywords:** distributed sensing, nanoparticles doped fibers, optical fibers, temperature monitoring, thermal ablation

## Abstract

Thermal ablation is achieved by delivering heat directly to tissue through a minimally invasive applicator. The therapy requires a temperature control between 50–100 °C since the mortality of the tumor is directly connected with the thermal dosimetry. Existing temperature monitoring techniques have limitations such as single-point monitoring, require costly equipment, and expose patients to X-ray radiation. Therefore, it is important to explore an alternative sensing solution, which can accurately monitor temperature over the whole ablated region. The work aims to propose a distributed fiber optic sensor as a potential candidate for this application due to the small size, high resolution, bio-compatibility, and temperature sensitivity of the optical fibers. The working principle is based on spatial multiplexing of optical fibers to achieve 3D temperature monitoring. The multiplexing is achieved by high-scattering, nanoparticle-doped fibers as sensing fibers, which are spatially separated by lower-scattering level of single-mode fibers. The setup, consisting of twelve sensing fibers, monitors tissue of 16 mm × 16 mm × 25 mm in size exposed to a gold nanoparticle-mediated microwave ablation. The results provide real-time 3D thermal maps of the whole ablated region with a high resolution. The setup allows for identification of the asymmetry in the temperature distribution over the tissue and adjustment of the applicator to follow the allowed temperature limits.

## 1. Introduction

Thermal ablation is a minimally invasive therapy that has been widely used for the treatment of tumors in different tissues, including liver, kidney, lung, bones [1], thyroid [2], and brain tissue [3]. The objective of the hyperthermic ablation is to destroy cancer cells by delivering heat directly to the tumor tissues using a needle-like applicator [4]. There are different types of thermal ablation techniques depending on the source of energy used to produce heat, such as radio-frequency (RF), microwave (MW), laser, and high-intensity focused ultrasound (HIFU) ablations [1]. In RF ablation, frictional heat is produced by an oscillating electrical current, which flows in the circuit created by two electrodes (on the applicator tip and on the skin) [1,5,6,7]. The MW ablation is based on an increase in the kinetic energy of the water molecules inside the tissue, which is induced by an electromagnetic field [4,8,9,10]. The laser ablation is achieved by the application of a laser to the tumor in order to produce light energy, which is then converted into heat [1,11,12]. The working principle of HIFU ablation is based on the application of ultrasound waves with a high intensity to heat the target tissue [1,13,14].

Nowadays, many researchers are investigating the application of nanoparticles in the localized thermal ablation procedure [15,16,17]. In particular, gold nanoparticles are actively used due to their bio-compatibility, bio-inertness, and unique tunable optical and electric properties, which are governed by the localized surface plasmon resonance phenomenon (LSPR) [18]. Due to LSPR, gold nanoparticles can absorb the scattering light and produce heat locally, which faciliatates a temperature increase at the desired area in a short period of time, preventing damage to surrounding healthy cells [19,20]. The size of the nanoparticles plays a crucial role in the efficiency of the ablation procedure and a size between 5–60 nm is widely used for thermal ablation therapy according to the cellular uptake and circulation in blood. The highest cellular uptake and higher LSPR effect observed for gold nanoparticles of size 20–60 nm [21], while smaller nanoparticles demonstrate better removal from the body [22].

The behavior of the tissue exposed to thermal ablation depends on the applied temperature. The tissue responds to a temperature of 41 °C by generating protective heat shock proteins, which increase the thermal resistance of the tissue in order to prevent thermal damage [1]. When the temperature is further increased to 42–46 °C, the tissue is more susceptible to irreversible damage, but the cells can still survive [23]. Temperatures higher than 46 °C cause cells death (the higher the temperature, the faster the death occurs). When the temperature of tissue reaches 60 °C, the plasma membrane melts, leading to almost instant cell death [1]. At temperatures greater than 105 °C, the tissue starts boiling, vaporizing, and carbonizing. In order to perform thermal ablation safely, the temperature must be maintained in the range of 50–100 °C throughout the process [23]. Therefore, it is crucial to use an accurate real-time temperature monitoring technique during the thermal ablation procedure.

Currently applied methods of temperature measurement can be classified into two groups: invasive techniques, which require insertion into the tissue, and non-invasive alternatives based on the imaging systems [24,25]. One of the invasive methods is based on the implementation of thermocouples, in which the sensing part is represented by two metallic wires joined in two junctions [25,26,27]. The main drawbacks of the thermocouples are an influence of the metallic components on the temperature propagation inside the tissue and the inability to measure temperature change over the entire ablated region [28]. Non-invasive methods are achieved by the use of medical imaging techniques based on computer tomography (CT) [29,30], ultrasound imaging [31,32], magnetic resonance tomography (MRT) [33,34], and others. The major advantages of the medical imaging methods are their non-invasiveness and ability to monitor temperature over the entire ablated region. Their limitations include a need to expose a patient to X-ray radiation for CT and the expense of MRT and CT scanners [24,28].

Another minimally invasive method used in thermal ablation is based on the application of fiber optic sensors (FOS) [24]. FOS became an important alternative for the aforementioned classical temperature monitoring techniques due to their miniature form factor, bio-compatibility, fast temperature response, and high resolution [28]. FOS sensors have no influence on the temperature propagation due to low heat conductivity, they are MR-compatible and can provide a cost-effective solution for thermal ablation application [35]. One of the popular FOS technologies used for this application is the Fiber Bragg Grating (FBG) sensor. FBG works on a particular wavelength, called the Bragg wavelength, which shifts proportionally to the applied temperature or strain. Several FBGs with different Bragg wavelengths can be incorporated into a single fiber by wavelength division multiplexing, thus, achieving a multiple-point sensing technology [35]. As a result, researchers have achieved real-time temperature monitoring with a spatial resolution of 10 mm [36], 6.5 mm [37], and 5 mm [38,39]. The spatial resolution can be further extended by a distributed sensing using a standard single-mode fiber (SMF). The operating principle of the distributed sensing is based on the Rayleigh backscattering, which occurs along the entire length of the fiber due to its small interior imperfections. The backscattered spectrum is a property of each fiber, which shows responses to the external stimulus, including temperature changes. Thus, temperature change at each point of the fiber can be retrieved by analysing the spectral shift of the backscattered signal [40]. The spatial resolution of the distributed sensing achieved by Gifford et al. is 20 µm [40] and by Macchi et al. is 200 µm [41].

Beisenova et al. have presented an approach for the multiplexing of the distributed sensing with the use of four high-scattering MgO-doped fibers as the sensing fibers, which are spatially separated by the SMF regions [28]. The setup has been applied for two-dimensional temperature monitoring of RF-ablated tissue with a pixel size of 2.5 mm × 5.0 mm [28]. In this work, the liver phantom of size 16 mm × 16 mm × 25 mm was exposed to MW ablation enhanced by gold nanoparticles of size 10 nm. According to Jelbuldina et al. [42], nanoparticle-mediated ablation in comparison with pristine ablation can dramatically increase the ablated region (even to more than double of the diameter) due to the variation of the thermoelectric properties of the tissue; however, the ablation shape is more irregular. Therefore, nanoparticle-enhanced ablation requires more precise temperature monitoring over the whole ablated region in three dimensions. Three-dimensional temperature measurement is achieved by increasing the number of multiplexed sensing fibers to twelve and arranging them around a heating applicator in two levels (inner and outer squares). A specific scaffold for precise insertion of the twelve fibers into the phantom has been designed for this experiment, which is essential to mitigate positioning errors. The fibers have been positioned 8 mm from each other and the measurements along the fibers have been taken every 2 mm, thus, the voxel size achieved by this setup is 2 mm × 8 mm × 8 mm.

## 2. Methodology

### 2.1. Experimental Setup

**Figure 1:**
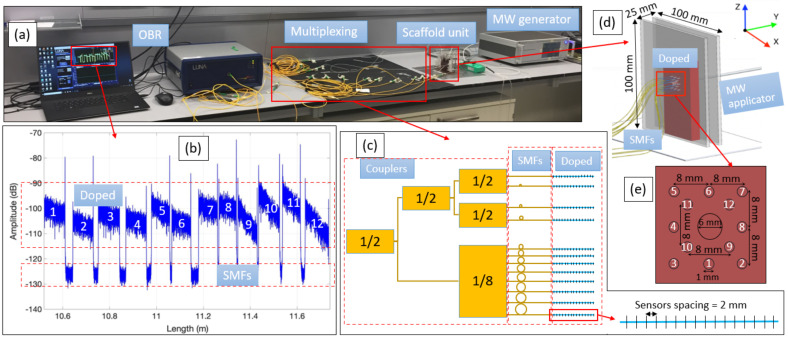
The experimental setup (**a**) consists of a Luna OBR interrogator with data acquisition software; Multiplexing setup for twelve high-scattering MgO nanoparticle-doped fibers spliced with single-mode fibers (SMFs) and connected through couplers (**b**,**c**); Scaffold (**d**) with a liver phantom and holes for holding an microwave (MW) applicator and twelve sensing fibers (**e**); MW generator to produce MW ablation.

As can be seen in Figure 1, the experiment has been performed on a swine liver phantom ablated by the MW generator (Leanfa Hybrid RF/MW generator). The liver has been pierced with twelve sensing optical fibers connected to the Optical Backscattering Reflectometry (OBR) interrogator (Luna OBR 4600, Luna Inc., Roanoke, VA, USA) to achieve 3D temperature monitoring.

### 2.2. Preparation of the Nanoparticle-Doped Liver Phantom

In the experiments, the thermal ablation has been enhanced by doping the tissue with the nanoparticles. Gold nanoparticles (GNPs, Sigma Aldrich, St. Louis, MO, USA) have been used because they have better stability in comparison with other nanoparticles and can considerably increase the heat above physiological temperature [43,44]. The stock solution of spherical gold nanoparticles of 10 nm in diameter stabilized in citrate buffer was dispersed in 0.2% agarose in a 1:1 ratio. The agarose solution was prepared according to the protocol done by Su et al. [45]. Finally, 100µL of diluted nanoparticles were taken from the prepared stock solution and administered ex vivo onto the surface of the tissue using a pipette. The thermal ablation procedure was conducted immediately after the injection of nanoparticles in order to prevent the leakage of nanoparticles. The nanoparticles were diluted in the agarose solution, which was not very dense in order to implement this technique further in vivo trials when the nanoparticles can be injected intravenously.

### 2.3. Preparation of the Multiplexing Setup for 3D Temperature Monitoring

Temperature monitoring of the thermal ablation has been conducted with the OBR instrument, the working principle of which is based on Rayleigh backscattering. The instrument measures the scattering of light from each infinitesimal section of the fiber and produces a spectrum of backscattered light as a function of position along the fiber (e.g., Figure 1b) [46,47,48]. The OBR instrument calculates the spectral shift of the fiber exposed to the external stimulus (temperature in this case) by dividing the spectrum into small Sections (5 mm in this experiment). Each section is cross-correlated with the corresponding section of the reference spectrum taken before the application of heat to the fiber. The spectral shift is converted to the wavelength shift, which is proportional to the applied temperature change [46]. The resolution of the measurement depends on the sensor spacing parameter, which has been set to 2 mm in this experiment (see Figure 1c).

The OBR equipment does not allow multiplexing because of the overlapping of the scattering patterns from several SMFs, but 3D temperature measurement requires multiple sensing fibers. Therefore, the high-scattering fibers were developed by doping the core of SMFs with MgO nanoparticles. The fabrication details and performance characteristics were discussed by Blanc et.al. [49,50]. MgO-doped fibers were spliced with SMF fibers with different lengths so that the length of each SMF is 1–2 cm longer than the length of the previous SMF plus MgO-doped fibers combined. Twelve MgO-doped fibers with SMF pigtails were connected to Luna OBR using a network of five couplers, as shown in Figure 1c. The scattering spectrum, given in Figure 1b, shows that twelve MgO-doped fibers with the scattering level in the range from −115 dB to −90 dB were separated by the lower scattering SMF regions in the range of −130 dB to −123 dB. The difference in the scattering levels of two types of fibers allows the regions corresponding to each of the twelve MgO-doped fibers to be clearly distinguished and, thus, temperature measurements to be obtained from each of them.

**Figure 2:**
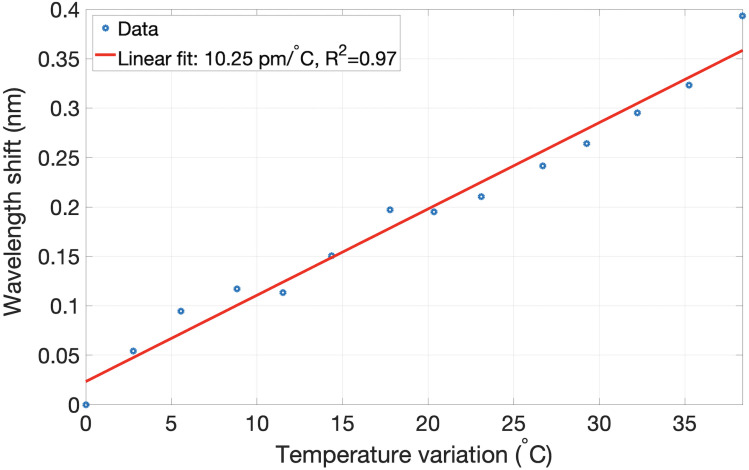
Wavelength shift recorded by the setup of twelve MgO-doped fibers with respect to temperature change.

In order to ensure that the wavelength shift recorded by OBR was proportional to the temperature applied to the twelve sensing fibers, the temperature calibration experiment was conducted. The twelve MgO-doped fibers were placed inside the water bath, together with a standard FBG sensor used as a reference. The water bath was placed on the heating plate, and its temperature was gradually increased from 21.8 °C to 60.8 °C. Each 3 °C, the temperature of the water was measured by FBGs connected to Micron Optics (si255, HYPERION), and simultaneously, the backscattering of the twelve fibers was recorded. Figure 2 illustrates the wavelength shift of the spectrum of all 12 fibers over the temperature recorded by FBG. According to the calibration results, the sensitivity of the setup is 10.25 pm/°C and the relationship is indeed linear.

### 2.4. Thermal Ablation Experiment

The nanoparticle-doped liver phantom was placed in the scaffold consisting of two plastic plates with dimensions of 100 mm × 100 mm × 5 mm. The plates were cut from a plexiglass sheet with a CO${}_{2}$ laser cutter machine (Epilog Fusion M2). The distance between the plates was adjusted to 25 mm and the liver was fixed inside the scaffold (see Figure 1d). The ablation was conducted by inserting an applicator of the MW generator into the middle of the liver through a central hole of the scaffold with a diameter of 6 mm. The applicator has a conic-shaped active electrode with a height of 1.0 cm. The MW generator was set to a power of 70 W and a frequency of 2.45 GHz for 70 s and then switched off. The temperature was monitored by twelve sensing fibers located around the applicator. The fibers were arranged, as shown in Figure 1e, in two levels: fibers 1–8 were located in the outer layer, further from the applicator, and fibers 9–12 were located in the inner layer, which was closer to the applicator. The fibers were inserted into the liver by placing a medical needle (21 G, Balton, Poland) inside the holes of the scaffold, passing each fiber through the needle, and then removing the needle. Temperature monitoring was conducted for 140 s in order to record the temperature during both the heating and cooling of the tissue. In total, 144 sensing points along the twelve fibers were used to monitor the temperature of the 16 mm × 16 mm × 25 mm volume of the liver. The resolution along the fibers was 2.0 mm (sensor spacing along each fiber) and perpendicular to the fibers, was 8.0 mm (distance between the fibers).

## 3. Results and Discussion

**Figure 3:**
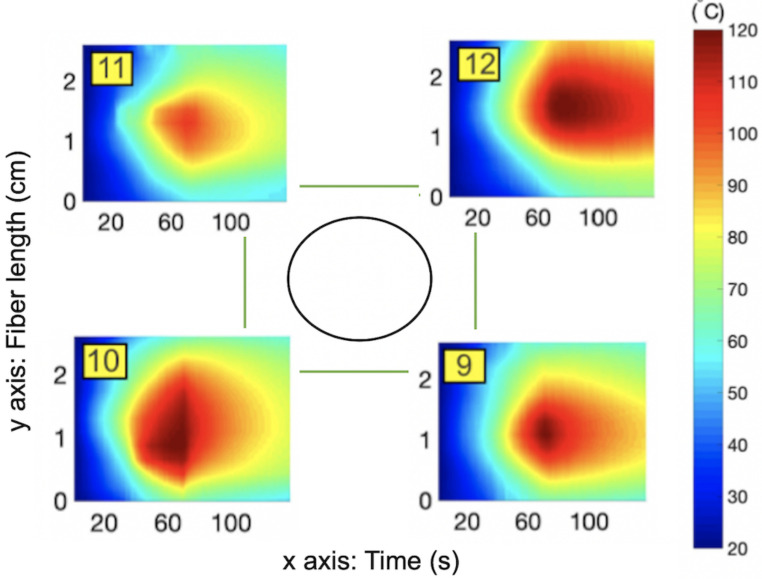
Temperature maps for the 9–12 fibers located closest to the applicator.

**Figure 4:**
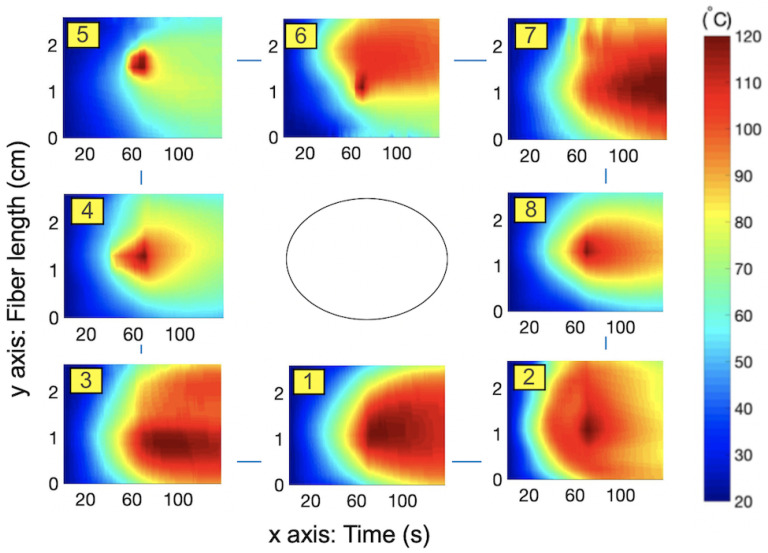
Temperature maps for the 1–8 fibers located furthest from the applicator.

**Figure 5:**
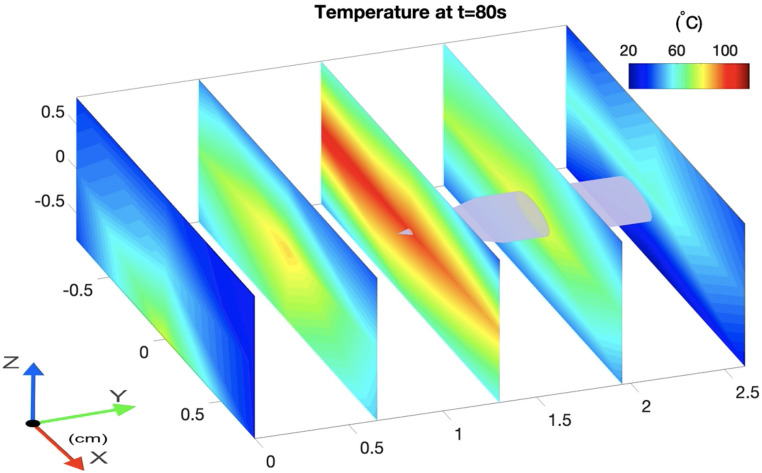
3D temperature profile of the scaffold during MW ablation (y-axis: parallel to the sensing MgO-doped fiber length and direction of the applicator, x and z-axes: horizontal and vertical directions of the fiber arrangement. (Video S1, MOV, 9.7 MB).

Thermal maps (temperature change over the ablated region over time), shown in Figure 3, Figure 4 and Figure 5, were obtained by interpolating the data from all twelve fibers using shading interp function in MATLAB, which resulted in such smooth temperature maps. Inner fibers (9–12) are shown in Figure 3 and outer fibers (1–8) are represented in Figure 4. The general pattern is common for all fibers: the temperature increased during the first half of the experiment because of the transmitted MW ablation energy and then slowly decreased after the thermal ablation ws stopped. Moreover, the highest temperature was detected by all of the fibers at their middle sensing regions (1.0–1.5 cm along y-axis) because the applicator tip was located in the middle of the scaffold. Inner fibers (9–12) detected a temperature increase earlier because of their closer location to the applicator tip. For example, inner fibers achieved a temperature of 90–100 °C after 40–50 s of ablation, while outer fibers detected this temperature after 50–60 s.

The heat propagated inside the tissue non-uniformly. For example, lower fibers 3, 1, and 10, were detected oto be at significantly higher temperatures than the corresponding upper fibers, 5, 6, and 11. On the right side, a slightly higher temperature was detected by upper fibers (7 and 12) than by respectful lower fibers (2 and 9). Asymmetry between the right and left sides was also present: right fibers 7, 8, and 12 were hotter than left fibers, 5, 4, and 11. The hottest region was the bottom-right corner and the coldest one was the upper-left corner. One of the possible reasons for such asymmetry is nonuniform temperature propagation over the tissue, which was amplified by the uneven distribution of gold nanoparticles inside the tissue. As previously mentioned, the use of the gold nanoparticles in thermal ablation results in a larger temperature increase and it is possible that the liquidised nanoparticles travelled to the bottom of the tissue during the procedure. This can explain the hotter lower regions of tissue. Another reason is a possible small shift of the applicator from the center. Both situations can occur during the real thermal ablation procedure. Therefore, it is important to monitor the temperature over the entire volume of ablated tissue. Three-dimensional temperature monitoring can accurately identify the spatial asymmetry, which can be used for reconstruction of the ablated tissue. It can serve as a visual tool for a clinician, who can adjust the applicator closer to the cooler regions during the procedure and achieve more uniform heating of the tissue.

**Figure 6:**
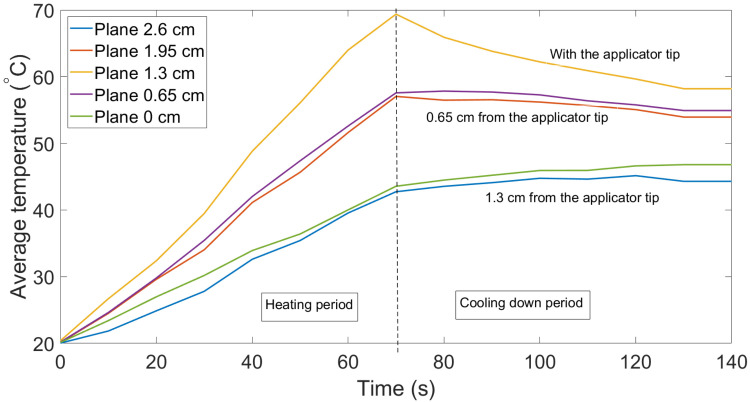
Change in the average temperature on the five cross-sections of the tissue along the y-axis: trial 1.

Figure 5 illustrates the caption of the 3D thermal profile of the swine liver at the 80th second (see Multimedia Data, Video S1). The video shows the temperature change over the duration of the experiment on the five selected cross-sections along the fibers and applicator (y-axis). The temperature change is shown every 10 s in order to reduce the file size, but the OBR is able to acquire the data at a frequency of 3 Hz. Each plane was created by interpolating the average temperature of four fibers located closest to the applicator and temperatures of the other eight fibers. The temperature increases throughout the tissue for the first half of the experiment and achieves a maximum temperature at approximately at 70–80 s. After that, the entire tissue started to gradually cool down because the MW generator was switched off. Since the applicator tip was located in the middle of the scaffold, the third cross-section, which was located on the y = 1.3 cm plane, experienced the highest temperatures during the whole experiment. This can also be seen in Figure 6, which illustrates the average temperature of all five cross-sectional planes. The highest average temperature was experienced by the middle plane with the applicator tip on it, followed by two neighboring planes located at distances 0.65 cm and 1.95 cm along the y axis.

**Figure 7:**
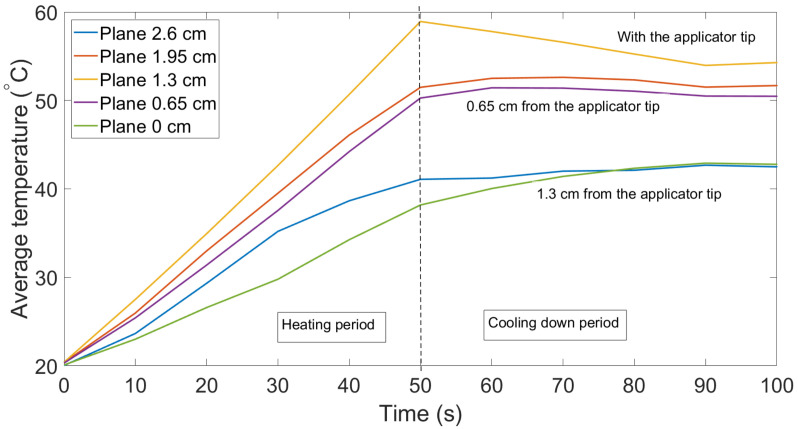
Change in the average temperature of the five cross-sections of the tissue along the y-axis: trial 2.

We performed the experiment with gold nanoparticles with several trials to validate that results were not affected by random events. Figure 7 indicates the change in the average temperature in the five layers where the duration of the experiment was 100 s and MW was stopped at the 50th s. Both trials were identical in temperature behavior, except for the fact that the highest temperature in Figure 7 was about 60 °C, which might change with the duration of the experiment.

## 4. Conclusions

In conclusion, we propose a distributed network that is able to perform a simultaneous scan of twelve fiber sensors for three-dimensional temperature monitoring during MW ablation of the porcine liver. The multiplexing of the twelve fibers is based on the higher Rayleigh backscattering of the MgO-doped fibers compared to the standard SMFs, which act as delays for the spacing of sensing regions. The twelve sensing regions, each 25 mm in length, were inserted through a plastic scaffold, which was used to hold the liver exposed to MW ablation. MW ablation was enhanced through ex vivo injection of gold nanoparticles in order to achieve better heat propagation. The results are presented as temperature planes of the scaffold unit, with a spatial resolution of 2 mm along the length of the MgO-doped fiber and of 8 mm in perpendicular directions. The setup has 144 sensing points for a tissue with a volume of 16 mm × 16 mm × 25 mm. The obtained thermal maps can serve as a real-time guidance method for nanoparticle-mediated MW ablation so that the applicator’s position or delivered power can be adjusted during the procedure to prevent tissue damage and achieve successful thermal ablation.

## Supplementary Materials

The following are available online at https://www.mdpi.com/1424-8220/21/3/828/s1, Video S1: The 3D temperature profile of the scaffold during MW ablation (y-axis: parallel to the sensing MgO-doped fiber length and direction of the applicator, x and z-axes: horizontal and vertical directions of the fiber arrangement.
